# ﻿*Impatiensliupanshuiensis* (Balsaminaceae), a new species from Guizhou, China

**DOI:** 10.3897/phytokeys.192.77269

**Published:** 2022-03-10

**Authors:** Tao-Hua Yuan, Yi Chen, Shuang Yu, Liu-Yi Ren, Rong-Xin Huang, Mei-Jun Li, Xin-Xiang Bai

**Affiliations:** 1 Forestry College, Guizhou University CN-5500252 Guiyang, China Guizhou University Guiyang China

**Keywords:** China, Flora of Guizhou, *Impatiens*, morphology, taxonomy

## Abstract

*Impatiensliupanshuiensis* (Balsamianceae), belonging to I.subgen.Impatiens, is recognised as a new species from Guizhou, China and it is described and illustrated. It is morphologically similar to *I.xanthocephala* W.W. Sm. in its yellow flowers, extremely small basal lobes on lateral united petals, broadly-dolabriform distal lobes and funnelform lower sepal. However, it is distinctive in the number of lateral sepals, teeth on the margin of lateral sepals, the recurvature of the dorsal petal, the number of lateral veins, the shape and size of the lamina and the type of lamina margin. A detailed description of the new species and colour photographs are provided. Its geographical distribution and morphology are also compared to similar species.

## ﻿Introduction

The genus *Impatiens*Linnaeus (1753: 937–938) (Balsaminaceae) contains over 1000 species distributed primarily in the Old World tropics and subtropics (Grey-Wilson 1980; Yu 2012). A few temperate outliers have also been identified in the Northern Hemisphere (Yu et al. 2016). Five conspicuous diversification centres in the Paleotropical Regions can be recognised: tropical Africa; Madagascar; southern India and Sri Lanka; the eastern Himalaya; and Southeast Asia area in the broad sense (Song et al. 2003; Yuan et al. 2004). In China, 349 species of *Impatiens* have been recorded (Yuan et al. in press), the overwhelming majority of them being distributed in the southwest region, including Yunnan, Sichuan, Tibet and Guizhou (Chen 2001; Yu 2012). Since 2021, according to our incomplete statistics, eight new species of *Impatiens* have been described from China (Gu et al. 2021; Liao et al. 2021; Peng et al. 2021a, b; Song et al. 2021a, b, c). In Guizhou, 61 species of *Impatiens* have been recorded (Peng et al. 2021a; Yu et al. 2021). Additionally, there is one other species of *Impatiens* from Guizhou currently in press (Ren et al. 2022).

It is well known that *Impatiens* species are notoriously difficult to classify (Hooker 1908; Grey-Wilson 1980) due to their semi-succulent stems and fleshy leaves, which make it difficult to dry and preserve herbarium specimens. As flowers are extremely fragile, they are difficult to separate and reconstruct from dried specimens, especially when critical floral parts are folded and coalesced. However, since the floral morphology is hyper-variable, the shape and size of petals and sepals are indispensable for identification. Thus, during field exploration, it is essential to make detailed records and separate the parts of the flowers on soft paper in situ.

In September 2016, during a botanical investigation in Zhongshan District, western Guizhou Province, China, we encountered an unusual species, which is only distributed in the *Fargesiaspathacea* Franch. (Poaceae) community in Xiaojiucaiping Mountain, the highest mountain of Guizhou (up to 2900 m alt.). The population of this unusual species is large. It resembles *Impatiensxanthocephala* W.W. Sm. (1920: 207–208) (Smith 1920) and *I.undulata* Y.L.Chen & Y.Q.Lu (1990: 23–25) (Chen and Lu 1990) in its yellow flowers, but the highly recurved dorsal petal is distinctive, allowing the anthers to be fully exposed. In September 2020 and August 2021, we revisited Xiaojiucaiping Mountain for a further field investigation to record morphological characters of the species. After careful examination of the relevant specimens and literature (Xiong and Luo 1989; Chen 2001; Chen et al. 2007; Yu 2012; Kuang et al. 2014; Kuang 2015; Luo and Deng 2015; Peng et al. 2021a), the authors decided that the species was hitherto undescribed and close to *I.xanthocephala* W.W. Sm. Hence, it is described here as a new species with a detailed description and illustrations.

## ﻿Materials and methods

The material for this study was mainly collected in the field at the type locality of the new species in 2020. Herbarium specimens were made carefully. The morphological characteristics of the new species were measured from fresh material and dried herbarium specimens by use of a ruler.

## ﻿Taxonomic treatment

### ﻿ Impatiens liupanshuiensis

Taxon classificationPlantaeEricalesBalsaminaceae

X.X.Bai & T.H.Yuan sp. nov.

8DDFD24B-A69F-5B3E-9622-5327B1969256

urn:lsid:ipni.org:names:77295796-1

Figs 1 , 2

#### Type.

China. Guizhou: Liupanshui City, Zhongshan District, Xiaojiucaiping Mountain, 2887 m alt., 26°51'8.76"N,104°41'33.67"E, 24 Aug 2021, *Xin Xiang Bai et al. BXX368* (holotype: GZAC!; isotype: PE!).

#### Diagnosis.

This species is similar to *Impatiensxanthocephala* W.W. Sm. in its yellow flowers, funnelform lower sepal, broadly-dolabriform distal lobes of lateral united petals and extremely small basal lobes, but it can be distinguished by having 2 (vs. 4) lateral sepals with ciliate (vs. entire) margin, dorsal petal recurved (vs. patent), lateral veins 8–10 pairs [vs. 2–3(4) pairs], apex of the distal lobes of the united petals retuse (vs. rounded), margin serrulate (vs. remotely crenate or shallowly undulate), lamina ovate-lanceolate or lanceolate (vs. ovate or ovate-oblong), 5–10.5 × 1.7–2.8 cm (vs. 1–2 × 0.8–1 cm) and lamina margin serrulate (vs. remotely crenate or shallowly undulate).

**Figure 1.**
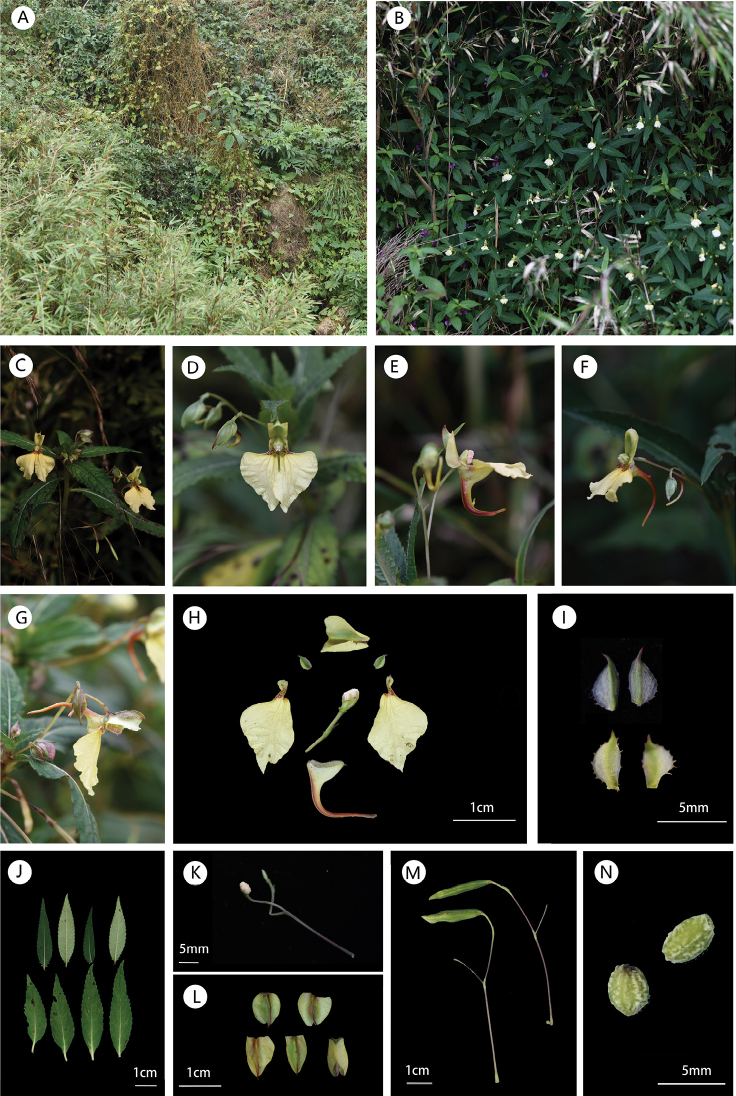
*Impatiensliupanshuiensis***A** habit **B** population **C, D** flower in face view **E, F** flower in lateral view **G** flower in dorsal view **H** flower dissected **I** lateral sepals (remotely denticulate on one or both sides) **J** leaves **K** stamens and pistils **L** different phases of the recurvature of the dorsal sepal **M** capsules **N** seeds. Photographs by X. X. Bai.

#### Description.

Perennial herb, 30–70 cm tall. Stem erect or procumbent basally, branched, upper part brown pubescent. Leaves alternate, petiolate, petioles 0.3–1 cm long. Lamina 5–10.5 × 1.7–2.8 cm, ovate-lanceolate or lanceolate, apex acuminate, base cuneate, with 1–3 pairs of basal glands, margin serrulate, teeth ending in setae, adaxial surface remotely puberulous on veins, abaxial surface glabrous, lateral veins 8–10 pairs. Inflorescences axillary, 2-flowered; peduncles 2–4 cm long, pedicels 1.5–2 cm long, bracteate above middle; bracts persistent, lanceolate, 2–4 mm long, margin denticulate. Flowers yellow, ca. 2 cm deep. Lateral sepals 2, obliquely ovate or ovate, inequilateral, 3–5 mm long, 2–3 mm wide, remotely denticulate on one or both sides, abaxial mid-vein slightly thickened, green, hyaline both sides, apex acuminate, aristate. Lower sepal funnelform, 0.5 cm deep excluding spur, mouth vertical, 0.5–0.9 cm wide, base abruptly contracted into an incurved spur, spur 1–1.5 cm long. Dorsal petal orbicular, 8–10 mm in diam., recurved at anthesis, abaxial mid-vein carinate, with a curved rostrum towards apex, base truncate, apex retuse. Lateral united petals 2-lobed, sessile, 13–18 mm long, basal lobes oblong, extremely small, 1–2 mm wide, distal lobes broadly-dolabriform, 1–1.3 cm long, 0.9–1 cm wide, apex retuse, base with reddish patches; auricle inflexed, small. Stamens 5, filaments linear, anthers obtuse. Ovary erect, fusiform, 5-carpellate. Capsule cylindrical, 1.5–2 cm long, seeds ellipsoid or ovoid, surface with warty protrusions.

**Figure 2.**
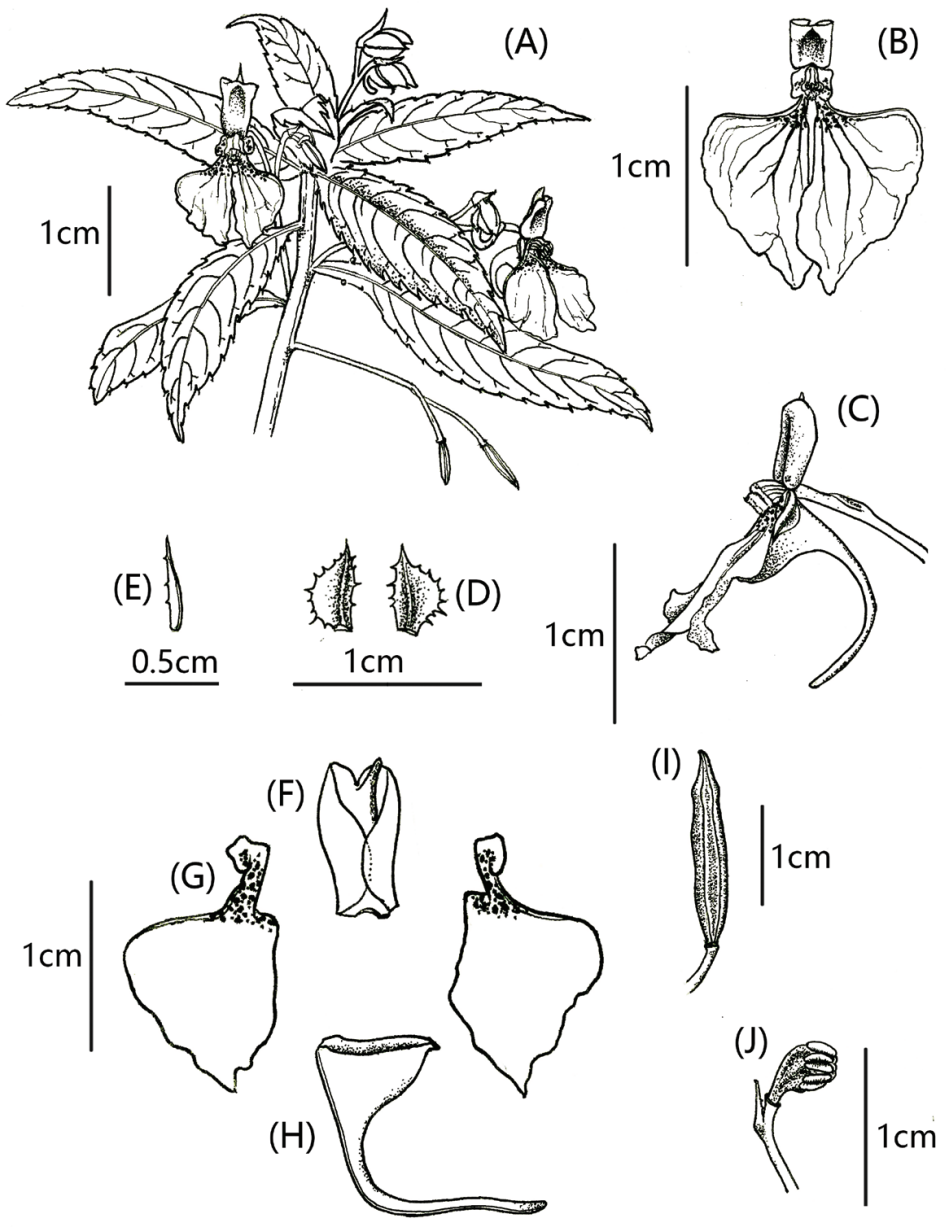
*Impatiensliupanshuiensis***A** plant **B** flower in face view **C** flower in lateral view **D** lateral sepals **E** bract **F** dorsal sepal **G** lateral united petal **H** lower sepal **I** capsule **J** stamens and pistils. (Drawn by Y. Chen from holotype and corresponding colour photographs).

#### Phenology.

This species was observed flowering from July to October and fruiting from September to November.

#### Distribution.

This species is currently known from only one population in Xiaojiucaiping Mountain, Zhongshan District, Liupanshui City, Guizhou, China. The population is large, with about 3000–3500 individuals.

#### Ecology.

This species was collected growing in *Fargesiaspathacea* shrubs on the side of the plank road at an elevation of 2730–2887 m in Xiaojiucaiping Mountain, which is the highest mountain in Guizhou. The main associated species were *F.spathacea*, *Impatienslecomtei*, *Sorbariaarborea* C.K. Schneid. (Rosaceae), *Pteridiumrevolutum* (Blume) Nakai (Dennstaedtiaceae), *Hypericumpatulum* Thunb. (Hypericaceae), *Eupatoriumlindleyanum* DC. (Asteraceae) and *Senecioscandens* Buch.-Ham. ex D. Don (Asteraceae).

#### Etymology.

The specific epithet ‘liupanshuiensis’ refers to the type locality, Liupanshui City, Guizhou, China. The Chinese name is given as “六盘水凤仙花”.

#### Conservation status.

*Impatiensliupanshuiensis* is currently known only from its type locality, Zhongshan District, Guizhou, China, where it is distributed in several places with about 3000–3500 individuals known in the population. As Xiaojiucaiping Mountain is a part of the Axilixi Landscape and Famous Scenery, it is an area exposed to significant human disturbance. The conservation status can be evaluated as Vulnerable (VU) C2a(ii), based on the IUCN Red List Categories and Criteria (IUCN 2019).

## ﻿Discussion

Yu et al. (2016) divided the genus Impatiens into two subgenera, subgen. Clavicarpa S.X. Yu ex S.X. Yu & Wei Wang and subgen. Impatiens, based on the number of carpels, the number of ovules per carpel, the shapes of the capsule, the number of pollen apertures and the pollen grain shape in polar view. In our study, *I.liupanshuiensis* is nested within subgen. Impatiens by its 5-carpellate ovary, cylindrical capsule and many ovules per locule. The new species is morphologically similar to *I.xanthocephala*, but *I.liupanshuiensis* has two lateral sepals with remote teeth on the margin, a more peculiar dorsal petal and more lateral veins and its flowers are shorter than its lamina. *I.liupanshuiensis* is also similar to *I.undulata*. To better distinguish the new species morphologically from *I.xanthocephala* and *I.undulata*, we list more details in Table 1.

**Table 1.** Comparison of morphological characters in *Impatiensliupanshuiensis*, *I.xanthocephala* (data from Smith 1920 and Chen et al. 2007) and *I.undulata* (data from Chen and Lu 1990 and Chen et al. 2007).

Characters/Species	*I.liupanshuiensis*	*I.xanthocephala*	*I.undulata*
Plant height	30–70 cm	10–16 cm	40–100 cm
Stem indumentum	upper part brown pubescent	glabrous	glabrous
Leaf indumentum	adaxial surface puberulous on veins	glabrous	glabrous
Lamina	ovate-lanceolate or lanceolate, 5–10.5 × 1.7–2.8 cm	ovate or ovate-oblong, 1–2 × 0.8–1 cm	ovate or ovate-orbicular, 2–7 × 1.5–5 cm
Lamina margin	serrulate	remotely crenate or shallowly undulate	undulate or obtusely crenate
Lateral veins	8–10 pairs	2–3(4) pairs	6–7 pairs
Flower size	small, ca. 2 cm deep	large, ca. 3.5 cm deep	small, 2 cm deep
Dorsal petal	orbicular, 8–10 mm, recurved, abaxial mid-vein carinate, a curved rostrum towards apex	suborbicular, 5–6 mm, abaxial mid-vein carinate at middle	suborbicular, 3–5 mm, abaxial mid-vein carinate
Lateral sepal	2, inequilateral, remotely denticulate on one side or both sides	4, outer 2 orbicular, inner 2 ovate, equilateral, entire	2, equilateral, entire
United lateral petals	apex of distal lobes retuse	apex of distal lobes rounded	apex of distal lobes retuse
Lower sepal	funnelform	funnelform	salverform
Anthers	obtuse	obtuse	acute

The distributions of these three species are geographically isolated from each other: *Impatiensliupanshuiensis* is confined to western Guizhou Province, *I.xanthocephala* is recorded in southwest Sichuan Province and *I.undulata* is recorded in central Sichuan Province and southern Chongqing Municipality (Yuan et al. in press).

## Supplementary Material

XML Treatment for Impatiens liupanshuiensis
